# Finding key genes (UBE2T, KIF4A, CDCA3, and CDCA5) co-expressed in hepatitis, cirrhosis and hepatocellular carcinoma based on multiple bioinformatics techniques

**DOI:** 10.1186/s12876-024-03288-7

**Published:** 2024-06-18

**Authors:** Yingai Zhang, Weiling Yu, Shuai Zhou, Jingchuan Xiao, Xiaoyu Zhang, Haoliang Yang, Jianquan Zhang

**Affiliations:** 1 Central Laboratory, Affiliated Haikou Hospital of Xiangya Medical College, Central South University, No.43 Renmin Road, Haikou, Hainan, 570208 China; 2 School of Life Sciences, Hainan University, No.58 Renmin Road, Haikou, Hainan, 570228 China; 3 Department of Chemotherapy, Affiliated Haikou Hospital of Xiangya Medical College, Central South University, No.43 Renmin Road, Haikou, Hainan, 570208 China; 4 Hepatobiliary surgery, Affiliated Haikou Hospital of Xiangya Medical College, Central South University, No.43 Renmin Road, Haikou, Hainan, 570208 China

**Keywords:** Hepatocellular carcinoma, Chronic active hepaititis B, Liver cirrhosis, Co-expressed genes, Bioinformatics

## Abstract

**Background:**

Hepatocellular carcinoma (HCC) is one of the most common cancers worldwide. Hepatitis B virus (HBV) is one of the major causes of liver cirrhosis (LC) and HCC. Therefore, the discovery of common markers for hepatitis B or LC and HCC is crucial for the prevention of HCC.

**Methods:**

Expressed genes for to chronic active hepaititis B (CAH-B), LC and HCC were obtained from the GEO and TCGA databases, and co-expressed genes were screened using Protein-protein interaction (PPI) networks, least absolute shrinkage and selection operator (LASSO), random forest (RF) and support vector machine - recursive feature elimination (SVM-RFE). The prognostic value of genes was assessed using Kaplan-Meier (KM) survival curves. Columnar line plots, calibration curves and receiver operating characteristic (ROC) curves of individual genes were used for evaluation. Validation was performed using GEO datasets. The association of these key genes with HCC clinical features was explored using the UALCAN database (https://ualcan.path.uab.edu/index.html).

**Results:**

Based on WGCNA analysis and TCGA database, the co-expressed genes (565) were screened. Moreover, the five algorithms of MCODE (ClusteringCoefficient, MCC, Degree, MNC, and DMNC) was used to select one of the most important and most closely linked clusters (the top 50 genes ranked). Using, LASSO regression model, RF model and SVM-RFE model, four key genes (UBE2T, KIF4A, CDCA3, and CDCA5) were identified for subsequent research analysis. These 4 genes were highly expressed and associated with poor prognosis and clinical features in HCC patients.

**Conclusion:**

These four key genes (UBE2T, KIF4A, CDCA3, and CDCA5) may be common biomarkers for CAH-B and HCC or LC and HCC, promising to advance our understanding of the molecular basis of CAH-B/LC/HCC progression.

## Background

Hepatocellular carcinoma (HCC) is the fourth leading cause of cancer mortality worldwide [1], and the survival rate of HCC is very low, with 5-year survival rates of 32.6%, 10.8% and 2.4% for local, regional, and distant diseases, respectively [2]. Nowadays, HCC is the sixth most common cancer in the world, and the global incidence is expected to increase significantly over the next 10 years [3, 4]. Although surgical resection, liver transplantation, hepatic artery chemoembolization (HACE), radiofrequency ablation, microwave ablation, interventional therapy, targeted therapy, and immunotherapy have been used to treat HCC in recent years, the 5-year survival rate after surgery has reached 50–70% [5–7]. Fortunately, technological development shows great potential to accurately find targets that lead to HCC. Studies on the mechanisms of HCC mainly focused on the pathogenesis of chronic hepatitis and liver cirrhosis (LC) [8, 9]. Previous researches has pointed out that HCC is generally referable to inflammation and other causative factors, which lead to persistent liver damage, thereby resulting in the activation of hepatic stellate cells and excessive deposition of extracellular matrix [10]. Chronic hepatitis may progress to liver fibrosis, LC, and even HCC that is a life-threatening disease posing a great threat to public health [10]. According to statistics, about 70% of HCC patients have hepatitis [11], 80–90% of HCC may be attributable to LC [12]. And among all types of hepatitis, hepatitis B virus (HBV) is one of the main causes of LC and HCC [13]. Therefore, in our study, we tried to find common biomarkers of chronic active hepaititis B (CAH-B), LC and HCC, and the early detection of the expression levels of them might serve to cure hepatitis and cirrhosis, thus reducing the incidence of HCC and improving the survival of HCC patients.

## Methods

### Data source

The gene expression matrix and clinical information about HCC, including 50 normal samples and 373 tumor samples, were obtained from the TCGA database (https://tcga-data.nci.nih.gov/tcga/), and data about CAH-B and LC were obtained from the GSE114783 dataset in the GEO database (https://www.ncbi.nlm.nih.gov/geo/), including 3 normal (NL) samples, 10 CAH-B samples, 10 LC samples and 10 HCC samples. The GSE89733 and GSE114564 datasets were used to validate gene expression profiles. Prognostic validation of HCC was performed by obtaining 240 corresponding clinical profiles from the ICGC database (https://dcc.icgc.org/releases/current/Projects).

### Weighted gene co-expression network analysis (WGCNA)

WGCNA is a powerful tool that identifies highly collaborative gene sets and helps to discover candidate biomarkers and therapeutic targets for cancer. Besides, it helps to discover biologically significant co-expressed gene modules by analyzing the interconnectivity of gene sets and their association with phenotypes, and to study the relationship between gene networks and disease [14]. In this investigation, CAH-B-, LC- and HCC-related gene modules were analyzed using WGCNA. The first step was to construct a sample-clustering tree based on the sample information. Next, a suitable soft threshold (β) was chosen based on the criteria of the scale-free network. Subsequently, a hierarchical clustering map was created to categorize genes with similar expression patterns into distinct modules. The correlation between modular signature genes and clinical features was then evaluated to identify relevant gene modules. Finally, scatter plots illustrating the correlations between key gene modules and clinical features were generated using the gg-plot package in R software.

### Screening and identification of hub genes

The Venn diagram of the key gene modules screened by TCGA and WGCNA was obtained using R software with the help of the gg-venn package. The GO and KEGG functional enrichment analyses of the intersecting gene set were carried out. After that, the Protein-protein interaction (PPI) network of the intersecting genes was analyzed using Metascape. The most important and tightest PPI expression cluster was screened using the MCODE plugin, and all genes in this key cluster were used for subsequent study. Next, these genes in the key cluster were further screened using the least absolute shrinkage and selection operator (LASSO), Random Forest (RF) and Support vector machines - Recursive Feature Elimination (SVM-RFE) models, and the 3 machine-learned intersection genes were taken as hub genes. The prognostic value of hub genes was assessed using Kaplan-Meier (KM) survival analysis. Finally, the differential expression of hub genes in NL, CAH-B, LC, and HCC was verified using the barplot package in R. The diagnostic value of hub genes was evaluated using R software to plot nomogram, calibration curve, and receiver operating characteristic (ROC) curve. The association of these four genes with HCC clinical features was explored using the UALCAN database (https://ualcan.path.uab.edu/index.html). CIBERSORT was used to quantify the proportion of immune cell infiltration and their correlation with candidate biomarkers.

## Results

### Screening of differentially expressed genes (DEGs)

**Fig. 1:**
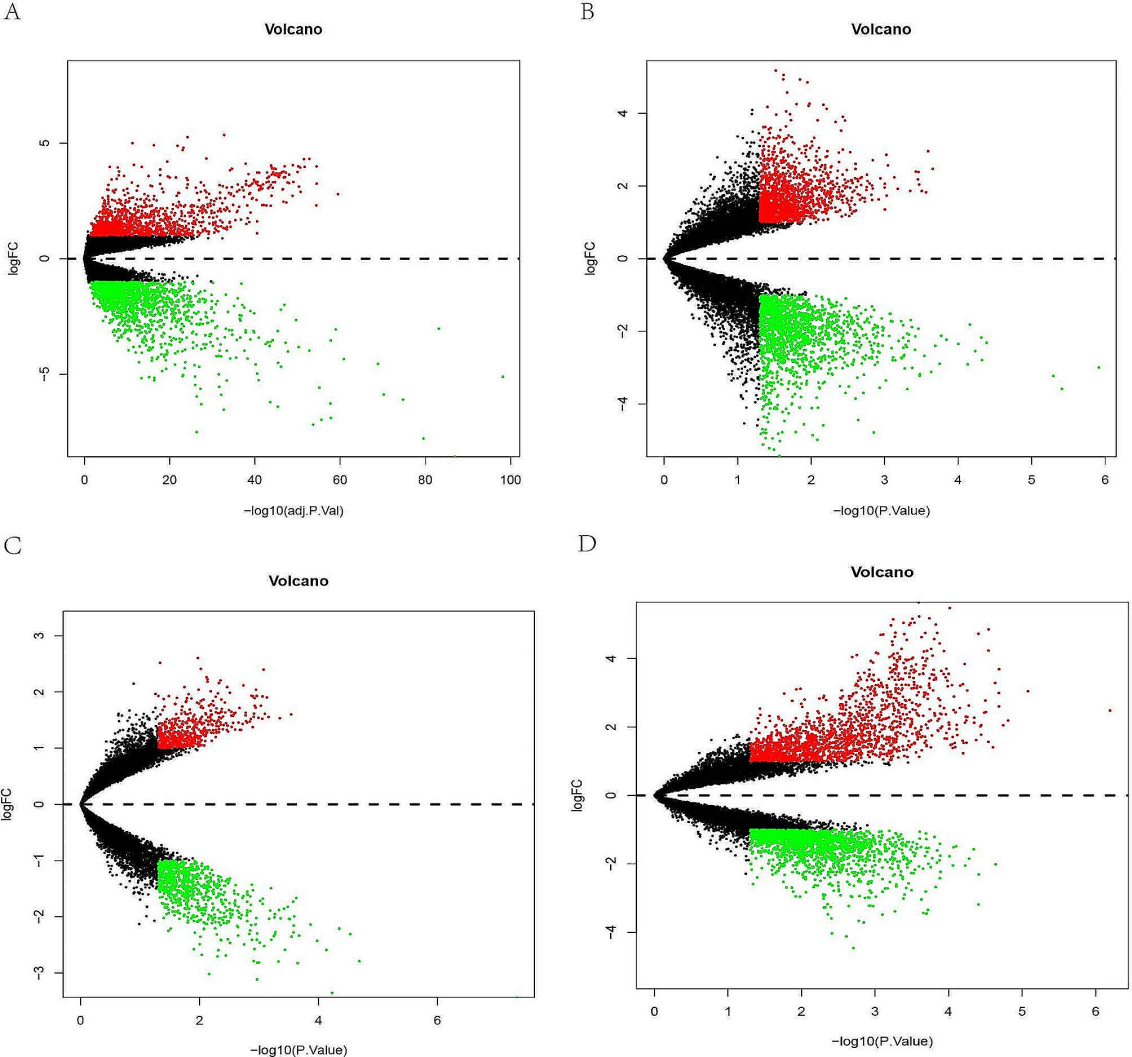
Volcano map of differential expression genes. Differential genes between normal and HCC **(A)**. Differential genes between normal and CAH-B **(B)**. Differential genes between normal and LC **(C)**. Differential genes between CAH-B and LC **(D)**

In our present study, DEGs in HCC tissue were obtained from the TCGA database. As shown in the volcano plot, the genes with adjusted (adj.) *p* < 0.05 and |logFC| > 1 were regarded as significant DEGs, and a total of 2834 DEGs (1275 up-regulated genes and 1559 down-regulated genes) were identified (Fig. 1A). We also obtained data related to CAH-B and LC from the GSE114783 dataset, and genes differentially expressed in NL versus CAH-B, NL versus LC, and CAH-B versus LC were demonstrated in volcano maps. More precisely, there were 2932 differential genes between NL and CAH-B with 1397 up-regulated and 1535 down-regulated, 1293 differential genes between NL and LC with 565 up-regulated and 728 down-regulated, and 3542 differential genes between CAH-B and LC with 1736 up-regulated and 1806 down-regulated (Fig. 1B, C, D). Genes met the screening conditions of *p* < 0.05 and |logFC| > 1 were regarded significantly differential.

### Weighted co-expression network construction and key module identification

**Fig. 2:**
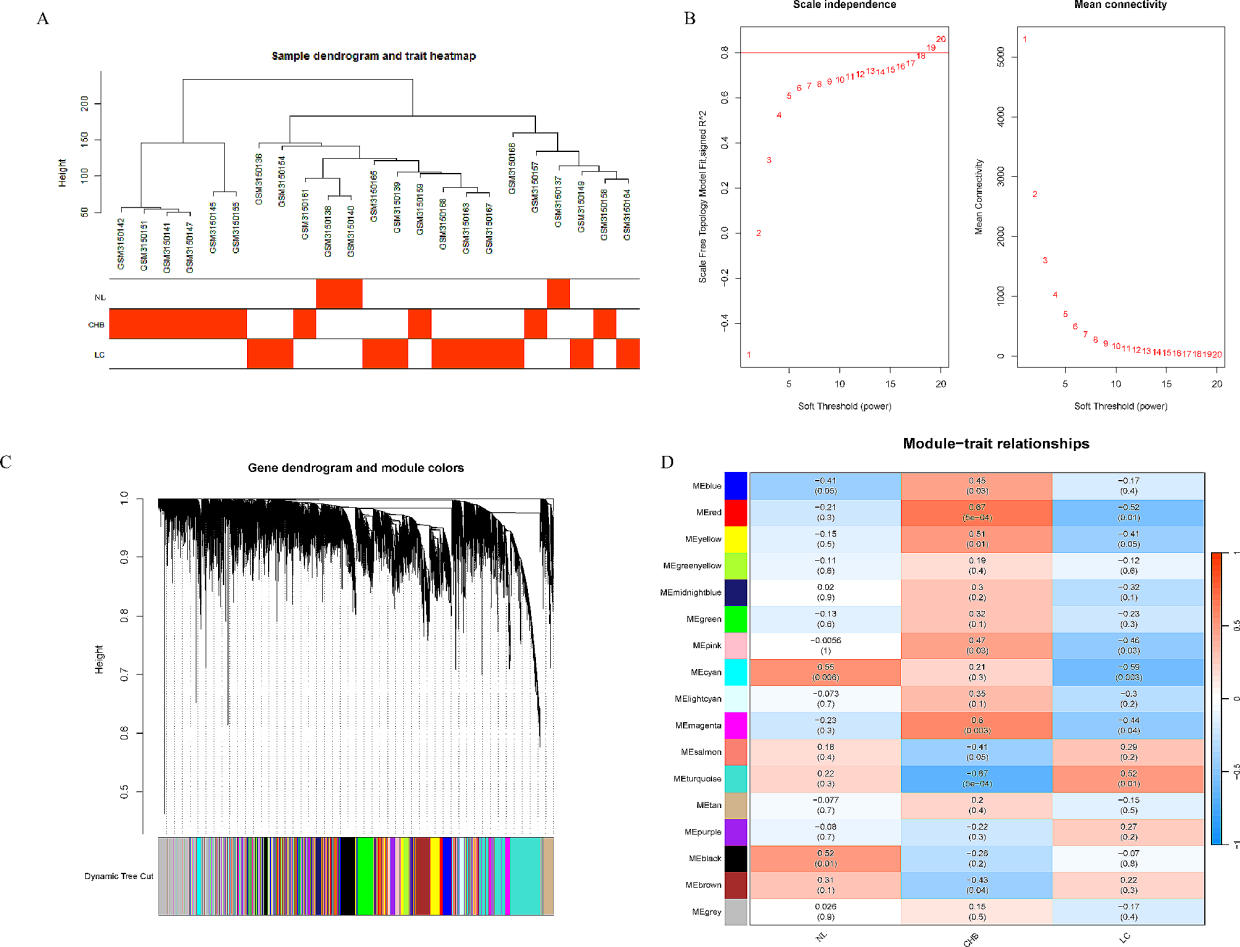
Determination of soft-threshold power in the WGCNA. Clustering dendrogram of 23 samples **(A)**. Analysis of the scale-free index and mean connectivity for various soft-threshold powers (β) **(B)**. Module hierarchical clustering diagram **(C)**. Heatmap of the correlation between the module eigengenes and clinical traits of NL, CHB and LC **(D)**

The data of gene expression about a total of 33 samples were obtained from the gene expression matrix GSE114783 by performing data preprocessing, and genes from these datasets with variance greater than all quartiles of variance were selected for further analysis. In addition, after excluding patients with incomplete clinical information, 10 HCC samples were excluded, and finally 23 clinical data (including 3 NL samples, 10 CAH-B samples, and 10 LC samples) were extracted for analysis. After removing outliers, the sample clustering tree was plotted based on the differentially expressed genes screened out above (Fig. 2A). As demonstrated in Fig. 2B, C, a scale-free network was constructed with a soft threshold of 19 (R^2^ = 0.82). Next, 17 modules were identified based on average hierarchical clustering and dynamic tree clipping (Fig. 2D). Among them, three modules, magenta, red and turquoise, were highly correlated with pathologic stages, and were selected for further analysis (Fig. 2E).

### Functional enrichment analysis of genes

**Fig. 3:**
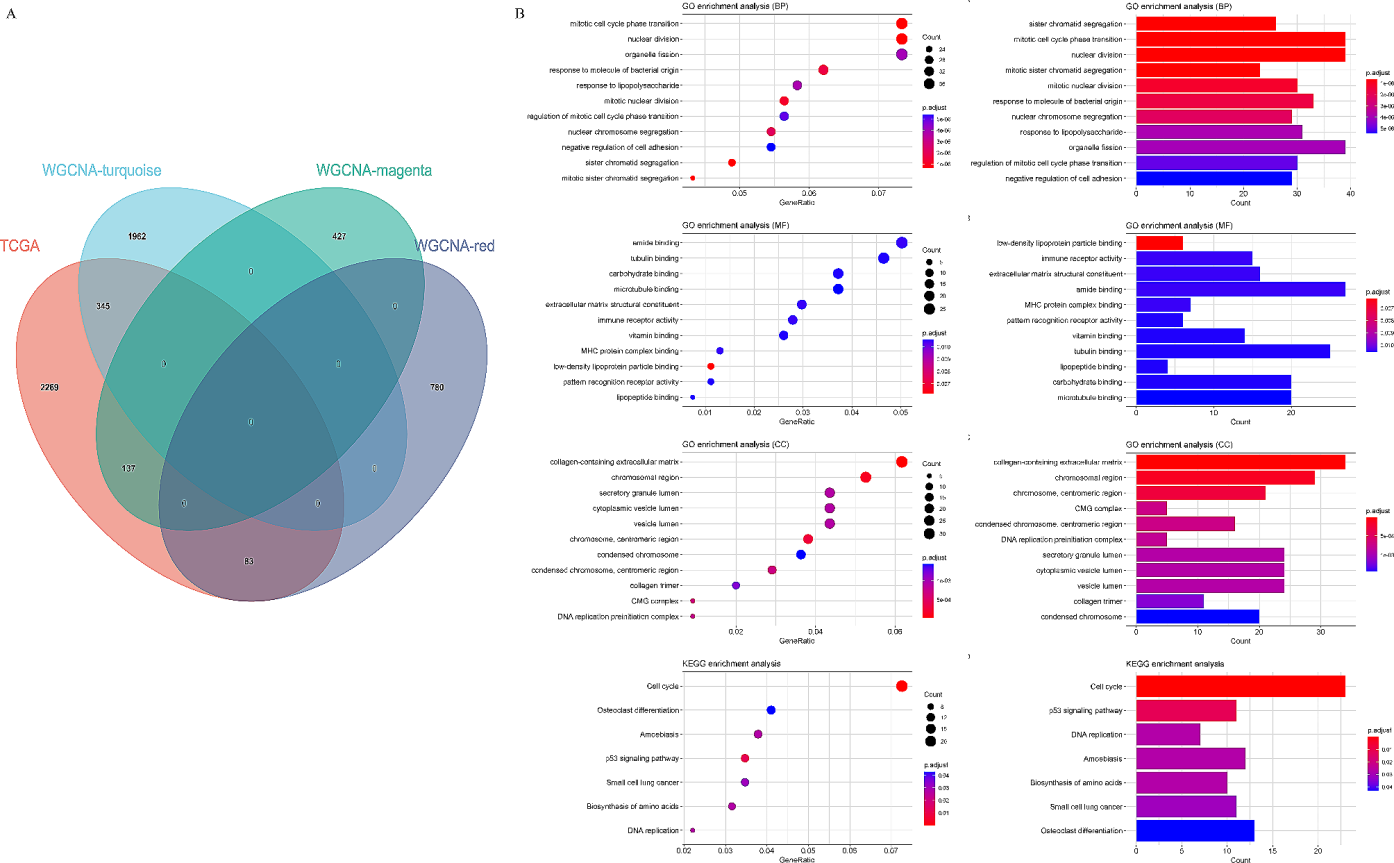
Gene function enrichment analysis. Intersection genes of TCGA and WGCNA magenta, red and turquoise modules **(A)**. GO and KEGG enrichment analysis of intersection genes **(B)**

Based on WGCNA analysis, three modules, magenta (564), red (863) and turquoise (2307), were highly correlated with pathologic stages of CAH-B and LC. A total of 2834 DEGs in HCC tissue were obtained from the TCGA database (Fig. 1A). To identify the key genes co-expressed in hepatitis, cirrhosis and hepatocellular carcinoma, the intersecting gene of the three modules obtained from WGCNA and TCGA differential genes was performed. We obtained 565 intersecting genes of TCGA with the three important modules mentioned above using a Wayne diagram (Fig. 3A). The GO and KEGG enrichment analyses were then carried out on the 565 intersecting genes (Fig. 3B), revealing that these genes were mainly associated with sister chromatid segregation, mitotic cell cycle phase transition, nuclear division (GO: Biological process); low-density lipoprotein particle binding, immune receptor activity, extracellular matrix structural constituent (GO: Molecular function); collagen-containing extracellular matrix, chromosomal region, chromosome centromeric region (GO: Cellular component); Cell cycle, p53 signaling pathway, and DNA replication (KEGG).

### Screening and identification of key genes

**Fig. 4:**
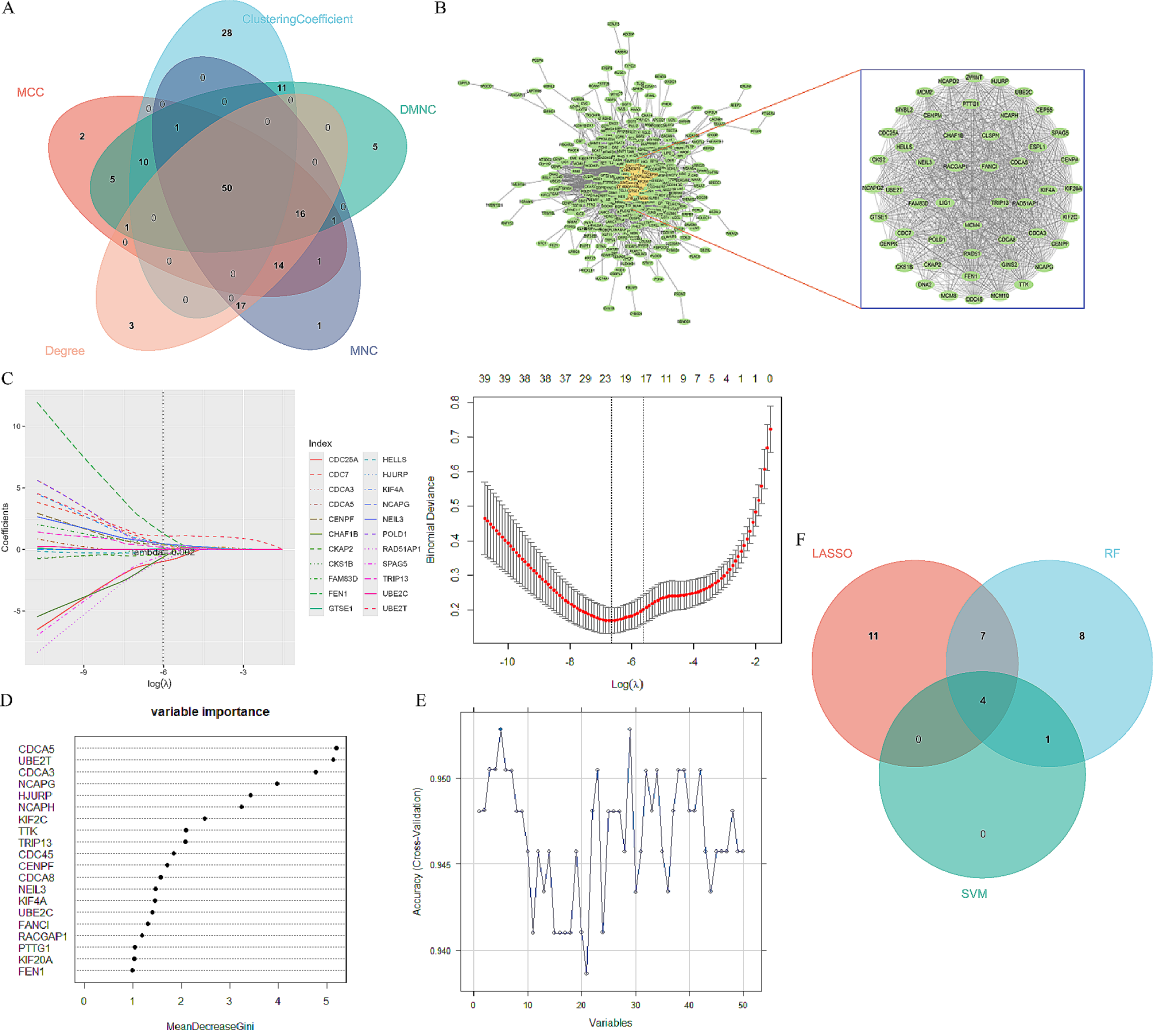
Screening and identification of Hub genes. Venn diagram shows the five algorithms of MCODE for the most closely linked gene clusters **(A)**. PPI network of Hub genes **(B)**. Coefficient profiles of candidate genes in LASSO model **(C)**. Coefficient profiles of candidate genes in RF algorithm **(D)**. Accuracy of candidate gene selection in SVM-RFE algorithm **(E)**. Venn diagram shows the overlap of characteristic genes of the three algorithms **(F)**

**Fig. 5:**
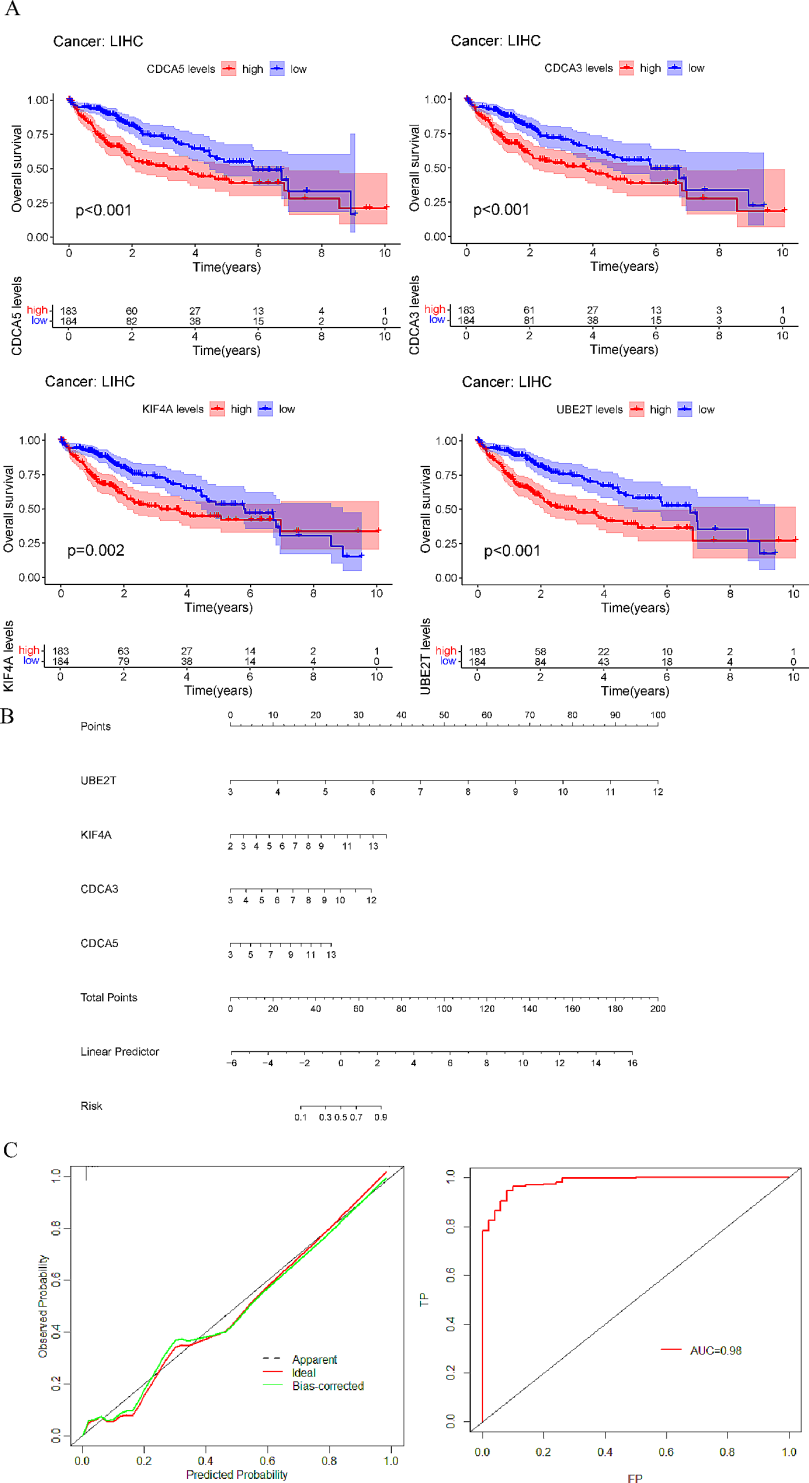
The prognostic and diagnostic value of Hub genes (UBE2T, KIF4A, CDCA3, and CDCA5) was analyzed. KM survival curves were used for prognostic analysis **(A)**. Nomogram **(B)** and ROC **(C)** curve for diagnostic efficacy

Based on the differentially expressed genes in CAH-B, LC and HCC acquired above, the co-expressed genes (565) of these three diseases were screened using PPI network, and PPI network was constructed (Fig. 4A, B). Moreover, the five algorithms of MCODE (ClusteringCoefficient, MCC, Degree, MNC, and DMNC) was used to select one of the most important and most closely linked clusters (the top 50 genes ranked). Finally, 50 genes were selected as out-of-hub genes. Next, LASSO regression model, RF model and SVM-RFE model were used to screen the critical genes simultaneously, and the intersection genes of the three algorithms were then obtained for subsequent analysis. First, the top 22 genes with the largest differential expression were screened based on the 50 screened hub genes for training optimization based on LASSO regression model, and 10-fold cross-validation was performed (Fig. 4C). Then, we obtained the importance measures of input variable using the RF algorithm and the top 20 genes with high importance measures (Fig. 4D). The SVM-RFE method was used to perform a round of elimination of the last few trait genes in the weight ranking of the training set, with five genes leaving (Fig. 4E). Finally, the intersection of the genes obtained from the three machine learning filters was again obtained using the Venn diagram, and finally four key genes (UBE2T, KIF4A, CDCA3, and CDCA5) were identified for subsequent research analysis (Fig. 4F). Followed by, the prognostic value of 4 genes was analyzed using KM survival curves, and it was found that these 4 genes had significant prognostic differences, and all were highly expressed with poor prognosis (Fig. 5A). Finally, we constructed Nomogram and performed calibration curve and ROC curve discriminations, and all three validation results were acceptable (AUC = 0.98, Fig. 5B and C). Consequently, these 4 genes were identified as the key genes for this study.

### Database validation

**Fig. 6:**
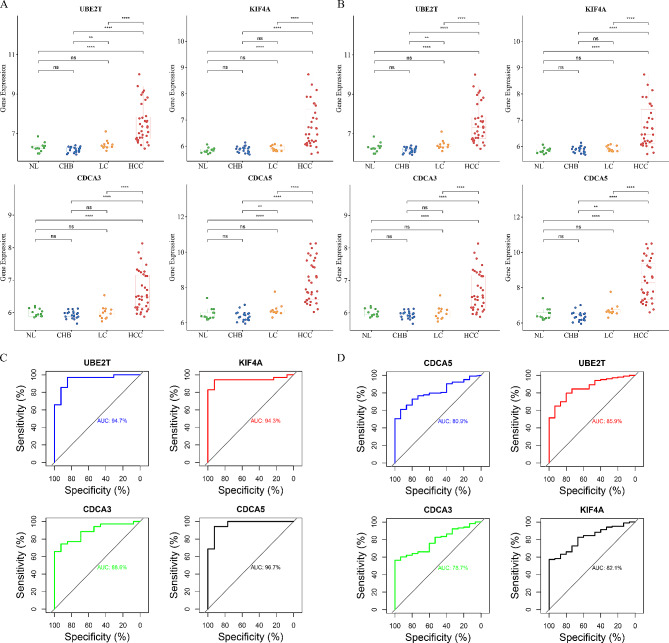
The expression and diagnostic efficacy of Hub genes in validation dataset. **(A)** Expression of Hub genes in GSE89377 dataset **(A)** and GSE114564 dataset **(B)**. ROC curve of Hub genes in GSE89377 dataset **(C)** and GSE114564 dataset **(D)**

The GSE89377 and GSE114564 datasets as validation dataset were introduced to verify the expression levels of these 4 genes (UBE2T, KIF4A, CDCA3, and CDCA5) in NL, CAH-B, LC, and HCC, and we found that the expression of the 4 hub genes was significantly different in CAH-B and HCC, LC and HCC (Fig. 6A and B). The diagnostic efficacy of UBE2T, KIF4A, CDCA3, and CDCA5 on the validation dataset were evaluated, and the AUCs were more than 0.78 (Fig. 6C and D), suggesting that the model exhibits high sensitivity and specificity in distinguishing among NL, CAH-B, LC, and HCC group.

**Fig. 7:**
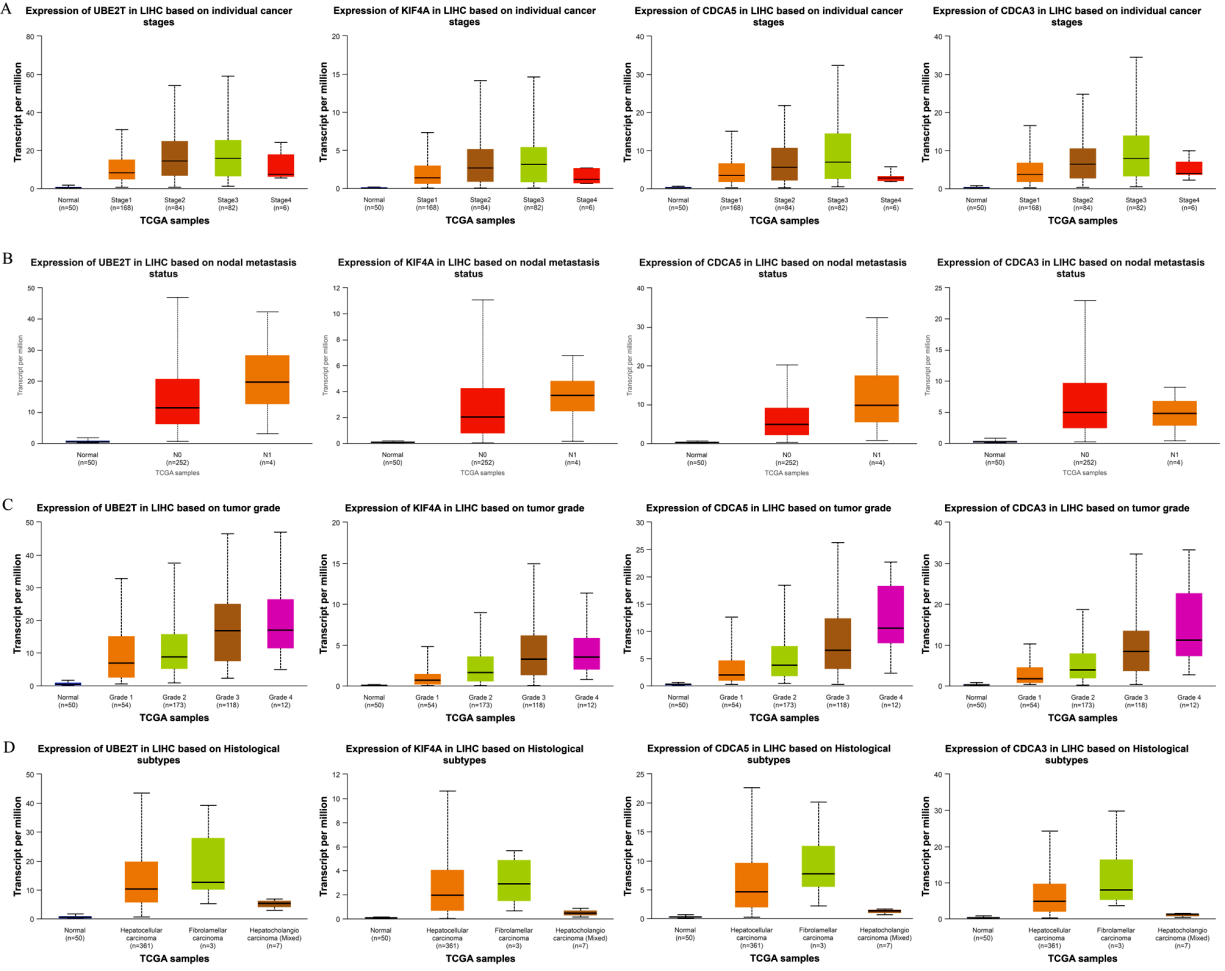
The expression levels of the hub genes—UBE2T, KIF4A, CDCA3, and CDCA5—were correlated with various clinical index of HCC. The expression levels of the hub genes with cancer stages **(A)**, nodal metastasis status **(B)**, tumor grade **(C)**, and histological subtypes **(D)**

Moreover, the association of these four genes with HCC clinical features was explored using the UALCAN database (https://ualcan.path.uab.edu/index.html). Our findings revealed that the expression levels of the hub genes—UBE2T, KIF4A, CDCA3, and CDCA5—were correlated with various clinical aspects of HCC, including cancer stages (Fig. 7A), nodal metastasis status (Fig. 7B), tumor grade (Fig. 7C), and histological subtypes (Fig. 7D).

**Fig. 8:**
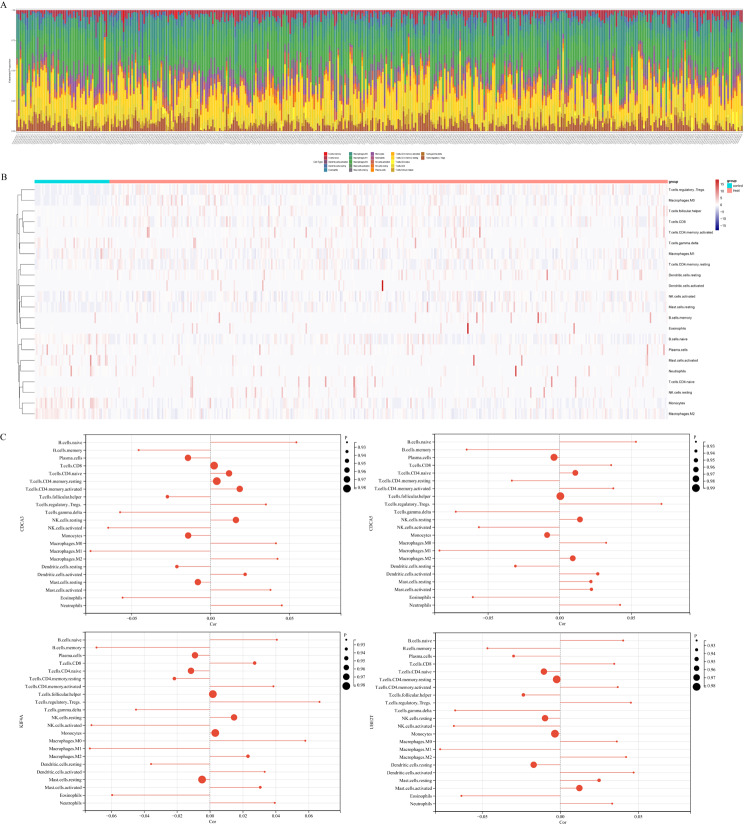
Landscape of immune cell infiltration in HCC. Histogram **(A)** and heatmap **(B)** plots of correlations among 22 immune cells. Correlation between four diagnostic markers and immune cell infiltration in HCC **(C)**

CAH-B, LC, and HCC represent a spectrum of inflammatory progression in liver disease. Therefore, we investigated the relationship between these four key genes and immune cell infiltration. The proportion of 22 types of immune cell infiltration in HCC was calculated (Fig. 8A and B). The association between four hub genes (UBE2T, KIF4A, CDCA3, and CDCA5) and 22 types of immune cell infiltration in HCC was presented in Fig. 8C.

## Discussion

Hepatitis and LC are the major causes of HCC [15, 16]. Chronic hepatitis may gradually develop into LC after being in a state of inflammation for a long time. LC can lead to a gradual decrease in liver function and cause HCC [17, 18]. Moreover, CAH-B is the most common form of hepatitis [13]. Therefore, the prevention of CAH-B and LC is an important measure for the timely treatment and prevention of HCC, and it is vital to find biomarkers common to CAH-B, LC and HCC.

In our study, the differentially expressed genes of CAH-B, LC and HCC were obtained from GEO and TCGA database. WGCNA (Weighted Gene Co-expression Network Analysis) is a systems biology method utilized for constructing and analyzing weighted co-expression networks to uncover the functional relationships among genes and identify gene modules that are highly interconnected and share similar expression patterns across conditions [19]. Based on WGCNA analysis, three modules, magenta (564), red (863) and turquoise (2307), were highly correlated with pathologic stages of CAH-B and LC. Combined with TCGA differential genes, 565 intersecting genes were obtained and used for GO and KEGG enrichment analyses. LASSO is a regression analysis method that performs both variable selection and regularization in order to enhance the prediction accuracy of statistical models by penalizing the absolute size of the coefficients [20]. Random Forest is an ensemble learning technique that constructs multiple decision trees during training and outputs the mode of the classes (classification) or mean prediction (regression) from these trees to provide a more accurate and robust prediction [21]. SVM-RFE is a powerful supervised learning algorithm used for both classification and regression tasks, which aims to find the optimal hyperplane that best separates different classes in the feature space or fits the regression function with minimal error [22]. Cross-validation with RF, LASSO and SVM-RFE algorithms was performed to mitigate the risk of overfitting. These three algorithms facilitated the selection of four overlapping hub genes (UBE2T, KIF4A, CDCA3, and CDCA5).

Ubiquitin coupling enzyme E2T (UBE2T) belongs to the ubiquitin coupling enzyme (E2) family and plays a crucial role in the ubiquitin proteasome pathway [23]. UBE2T has been reported to play a crucial role in protein ubiquitination, and it is essential for regulating many biological processes, including inflammation, immune response, cell division, and cell proliferation [24]. UBE2T overexpression facilitates the growth, proliferation, and invasion of colorectal cancer cells and suppresses apoptosis [25]. UBE2T gene, located in 1q32.1, has been reported to be up-regulated in HCC to promote HCC progression [26]. In HCC, UBE2T was identified as a novel target for SENP1 and a potential role of SENP1 in promoting UBE2T expression and deSUMOylation was identified [27]. However, no study reported the role of UBE2T in chronic active hepatitis B and liver cirrhosis. In our study, UBE2T was abnormally expressed in CAH-B versus LC, CAH-B versus HCC, and LC versus HCC.

Human cell division cycle-associated 5 (CDCA5) is required for sister chromatid condensation in S and G2 phases, promotes complex-dependent ubiquitination degradation in late G1 phase, phosphorylates and dissociates from chromatids in proprometaphase [28]. In addition, CDCA5 was found to be significantly upregulated in human tumor tissues, including HCC [29]. CDCA5, transcribed by E2F1, might promotes oncogenesis by enhancing cell proliferation and inhibiting apoptosis via the AKT Pathway in HCC [30]. A twenty gene-based gene (including CDCA5) set variation score reflected the pathological progression from cirrhosis to hepatocellular carcinoma [31]. However, no study reported the role of CDCA5 in chronic active hepatitis B and liver cirrhosis. In our study, CDCA5 was abnormally expressed in CAH-B versus LC, CAH-B versus HCC, and LC versus HCC.

Cell division cycle-associated protein-3 (CDCA3) controls translation to influence the cell cycle in the G1 phase as cells cannot transfer from the G2 to M phase without CDCA3 expression [32]. CDCA3 is frequently upregulated in the tumor tissues and is associated with oncogenic properties in several cancers, including HCC. The expression of CDCA3 was upregulated by E2F4 to promote the proliferation of HCC [33]. The CDCA3 mRNA was found to be the intracellular target of miR-145, inhibiting proliferation and migration of liver cancer cell lines [34]. However, no study reported the role of CDCA3 in chronic active hepatitis B and liver cirrhosis. In our study, CDCA3 was abnormally expressed in CAH-B versus LC, CAH-B versus HCC, and LC versus HCC.

The gene expressing kinesin family member 4 A (KIF4A) is located at Xq13.1 in the human genome, and the 140-kDa protein is mainly located in the nucleus [35]. KIF4A was observed significantly higher mRNA and protein expression in HCC tissues, and the mRNA expression of KIF4A correlated markedly with individual cancer stages and tumor grades [36]. Upregulation of KIF4A enhanced cell proliferation via activation of Akt signaling and predicted a poor prognosis in HCC [37]. Hepatitis B virus might upregulate the expression of KIF4A, and the activation of the gene expression of KIF4A increased in a pHBV1.3 concentration‑dependent manner [38]. In our study, KIF4A was abnormally expressed in CAH-B versus LC, CAH-B versus HCC, and LC versus HCC. The role of these genes (UBE2T, CDCA3, and CDCA5) in the progression of hepatitis, cirrhosis and hepatocellular carcinoma remains to be further investigated. Our findings, combined with previous studies, suggested that most of the key genes we have screened were strongly associated with the development of CAH-B, LC and HCC. In addition, the expression of key genes differed significantly between CAH-B and HCC, as well as LC and HCC, and the expression levels showed a progressive increase. These genes may have pro-inflammatory effects and indirectly promote the conversion of CAH-B and LC to HCC. Hence, changing their expression levels or activity may play a role in suppressing inflammation and cirrhosis, thereby reducing the incidence of HCC. The expression of UBE2T, KIF4A, CDCA3, and CDCA5 was related to the infiltration of immune cells and gene markers of immune infiltrating cells. These results provide evidence that UBE2T, KIF4A, CDCA3, and CDCA5 is involved in immune escape and immunosuppression in the tumor microenvironment.

Although this study provided a new insight into finding the common biomarkers for CAH-B, LC and HCC and the early prevention of tumorigenesis, it still has some limitations. We are predicting based on bioinformatics that genes may be strongly associated with hepatitis, LC and HCC, but the sample size of these data is limited, only CAH-B samples from hepatitis were screened. Besides, the expression of these candidate genes in clinical samples are needed to further confirm our findings. In addition, their correlation with information (HBV infection status such as HBe, HBV-DNA, HBV genotype, fibrosis or chronic liver injury state, and HCC staging, pathological information) was not explored. In the follow-up study, we will collect samples of CAH-B, LC, and HCC patients and their clinical information to examine the expression of these genes and their association.

## Conclusion

These four key genes (UBE2T, KIF4A, CDCA3, and CDCA5) may be common biomarkers for CAH-B and HCC or LC and HCC, promising to advance our understanding of the molecular basis of CAH-B/LC/HCC progression.

## Data Availability

No datasets were generated or analysed during the current study.

## Abbreviations

HCC: Hepatocellular carcinoma
HACE: Hepatic artery chemoembolization
HBV: Hepatitis B virus
LC: Liver cirrhosis
CAH-B: Chronic active hepaititis B
NL: Normal
WGCNA: Weighted gene co-expression network analysis
LASSO: Least absolute shrinkage and selection operator
RF: Random forest
SVM-RFE: Support vector machine - recursive feature elimination
KM: Kaplan-Meier
ROC: Receiver operating characteristic
DEGs: Screening of differentially expressed genes
UBE2T: Ubiquitin coupling enzyme E2T
CDCA5: Cell division cycle-associated 5
CDCA3: Cell division cycle-associated protein-3
KIF4A: Kinesin family member 4 A
PPI: Protein-protein interaction
